# Ionic mechanisms of spinal neuronal cold hypersensitivity in ciguatera

**DOI:** 10.1111/ejn.13098

**Published:** 2015-11-13

**Authors:** Ryan Patel, Nicola L. Brice, Richard J. Lewis, Anthony H. Dickenson

**Affiliations:** ^1^ Department of Neuroscience, Physiology and PharmacologyUniversity College LondonGower StreetLondonWC1E 6BTUK; ^2^ Takeda Cambridge LtdCambridgeUK; ^3^ Institute for Molecular BioscienceThe University of QueenslandSt LuciaQLDAustralia

**Keywords:** ciguatoxin, dorsal horn, *in vivo* electrophysiology, Na_v_1.8, rat, TRP channels

## Abstract

Cold hypersensitivity is evident in a range of neuropathies and can evoke sensations of paradoxical burning cold pain. Ciguatoxin poisoning is known to induce a pain syndrome caused by consumption of contaminated tropical fish that can persist for months and include pruritus and cold allodynia; at present no suitable treatment is available. This study examined, for the first time, the neural substrates and molecular components of Pacific ciguatoxin‐2‐induced cold hypersensitivity. Electrophysiological recordings of dorsal horn lamina V/VI wide dynamic range neurones were made in non‐sentient rats. Subcutaneous injection of 10 nm ciguatoxin‐2 into the receptive field increased neuronal responses to innocuous and noxious cooling. In addition, neuronal responses to low‐threshold but not noxious punctate mechanical stimuli were also elevated. The resultant cold hypersensitivity was not reversed by 6‐({2‐[2‐fluoro‐6‐(trifluoromethyl)phenoxy]‐2‐methylpropyl}carbamoyl)pyridine‐3‐carboxylic acid, an antagonist of transient receptor potential melastatin 8 (TRPM8). Both mechanical and cold hypersensitivity were completely prevented by co‐injection with the Na_v_1.8 antagonist A803467, whereas the transient receptor potential ankyrin 1 (TRPA1) antagonist A967079 only prevented hypersensitivity to innocuous cooling and partially prevented hypersensitivity to noxious cooling. In naive rats, neither innocuous nor noxious cold‐evoked neuronal responses were inhibited by antagonists of Na_v_1.8, TRPA1 or TRPM8 alone. Ciguatoxins may confer cold sensitivity to a subpopulation of cold‐insensitive Na_v_1.8/TRPA1‐positive primary afferents, which could underlie the cold allodynia reported in ciguatera. These data expand the understanding of central spinal cold sensitivity under normal conditions and the role of these ion channels in this translational rat model of ciguatoxin‐induced hypersensitivity.

## Introduction

Cold hypersensitivity can occur after nerve injury and is especially prevalent in patients with chemotherapy‐induced neuropathy. Ciguatera is a common ichthyosarcotoxism characterized initially by gastrointestinal disturbances (vomiting, nausea and diarrhoea) followed by neurological disturbances (paraesthesiae and dysaesthesiae in the extremities, pruritus and cold allodynia). As with some patients with neuropathy, innocuous cold temperatures elicit sensations of intense stabbing, pricking and burning pain (Ochoa & Yarnitsky, 1994; Berglund *et al*., 2002; Zimmermann *et al*., 2013). Muscle weakness, fatigue, joint pain and mood disturbances are also prominent, with severe cases presenting with cardiovascular disturbances and respiratory depression. The gastrointestinal effects are typically transient (1–5 days), whereas neurological symptoms can persist for weeks to many months (Lewis, 2006).

Ciguatera is caused by ciguatoxins, polycyclic polyether toxins produced by dinoflagellates of the *Gambierdiscusare* genus, that accumulate in the food chain (Lewis, 2006). Ciguatoxins are potent activators of sodium channels acting through neurotoxin site 5, also targeted by the structurally related brevetoxins (Bidard *et al*., 1984; Cestele & Catterall, 2000). *In vitro* electrophysiological studies of the effect of ciguatoxins on tetrodotoxin‐sensitive and tetrodotoxin‐resistant voltage‐gated sodium channels reveal a hyperpolarizing voltage shift in activation/inactivation, and an increased rate of recovery from inactivated states, respectively (Strachan *et al*., 1999; Yamaoka *et al*., 2009), likely leading to channel activation at resting membrane potentials, ongoing activity in primary sensory afferent fibres, axonal swelling and elevation of intracellular calcium (Molgo *et al*., 1993; Vetter *et al*., 2012; Mattei *et al*., 2014). Ciguatoxins also inhibit delayed rectifier and A‐type potassium currents in dorsal root ganglia neurones, resulting in further increases in neuronal excitability (Birinyi‐Strachan *et al*., 2005).

Sensory disturbances, like other neurological symptoms, could arise from central neurotoxic effects (Zhang *et al*., 2013); however, intradermal injection of 0.1–10 nm Pacific ciguatoxin (P‐CTX)‐1 in human subjects displays the somatosensory hallmarks of systemic ciguatera poisoning, namely pruritus at low concentrations and cold allodynia at higher concentrations, indicating direct actions on primary sensory afferents (Zimmermann *et al*., 2013). In a mouse model, intraplantar P‐CTX‐1 and P‐CTX‐3 induced spontaneous flinching and increased nocifensive behaviours in response to cold temperatures (Vetter *et al*., 2012; Zimmermann *et al*., 2013). Cold hypersensitivity appears to be the predominant sensory disturbance in this model, with no mechanical or thermal hypersensitivity apparent, and distinct populations of peptidergic dorsal root ganglia neurones involving tetrodotoxin‐sensitive and tetrodotoxin‐resistant voltage‐gated sodium channel components were proposed to underlie this (Vetter *et al*., 2012).

P‐CTX‐2 is a less‐oxidized form of P‐CTX‐1 that exists as a diastereomer of P‐CTX‐3 and has not been extensively characterized *in vivo* (Lewis *et al*., 1993). Previous mouse studies have examined peripheral and supra‐spinal mechanisms that may underlie cold allodynia reported in patients with ciguatera and in a surrogate model in healthy volunteers (Vetter *et al*., 2012; Zimmermann *et al*., 2013). To extend previous *in vitro* studies, the present study aimed to examine the back‐translation of this surrogate model by utilizing electrophysiological recordings of rat dorsal horn neurones receiving convergent input within an integrated physiological system, which code normal and experimental pain conditions in a remarkably similar way to human subjects (O'Neill *et al*., 2015). We wished to identify spinal neuronal mechanisms and the pharmacological basis of cold sensitivity and ciguatoxin‐induced cold hypersensitivity.

## Materials and methods

### Animals

Male Sprague‐Dawley rats (8–10 weeks old, 250–300 g) were used for electrophysiological experiments (Biological Services, University College London, UK). Animals were group housed on a 12 h/12 h light/dark cycle, and food and water were available *ad libitum*. The temperature (20–22 °C) and humidity (55–65%) of holding rooms were closely regulated. All procedures described here were approved by the UK Home Office, adhered to the Animals (Scientific Procedures) Act 1986, and were designed in accordance with IASP (International Association for the Study of Pain) ethics guidelines (Zimmermann, 1983).

### In vivo *electrophysiology*

*In vivo* electrophysiology was conducted as previously described (Patel *et al*., 2014b). Animals were anaesthetized with 3.5% v/v isoflurane delivered in a 3 : 2 ratio of nitrous oxide and oxygen. Once areflexic, a tracheotomy was performed and rats were subsequently maintained on 1.5% v/v isoflurane for the remainder of the experiment. Rats were secured in a stereotaxic frame and a laminectomy was performed to expose the L4–L5 segments of the spinal cord. Extracellular recordings were made from deep dorsal horn wide dynamic range lamina V/VI neurones with receptive fields on the glabrous skin of the toes using parylene‐coated tungsten electrodes (A‐M Systems, Sequim, WA, USA). Neurones were characterized from depths relating to deep dorsal horn laminae (Watson *et al*., 2009), and were selected on the basis of responses to dynamic brushing, and noxious mechanical and thermal stimulation.

The electrical stimulation of wide dynamic range neurones was delivered transcutaneously via needles inserted into the receptive field. A train of 16 electrical stimuli (2 ms pulses, 0.5 Hz) was applied at three times the threshold current for C‐fibre activation. Responses evoked by Aβ‐fibres (0–20 ms), Aδ‐fibres (20–90 ms) and C‐fibres (90–350 ms) were separated and quantified on the basis of latency. Neuronal responses occurring after the C‐fibre latency band were classed as post‐discharge. The input and wind‐up were calculated as

input=(action potentials evoked by first pulse)×total number of pulses(16)

wind‐up=(total action potentials after 16 train stimulus)−input

The receptive field was also stimulated using a range of natural stimuli [brush, von Frey filaments (2, 8, 15, 26 and 60 g) and heat (35, 42, 45 and 48 °C)] applied over a period of 10 s per stimulus and the evoked response was quantified. The heat stimulus was applied with a constant water jet onto the centre of the receptive field. Acetone (Sigma, UK) and ethyl chloride (100 μL) (Miller Medical Supplies, Newport, UK) were applied to the receptive field, described previously as an evaporative innocuous cooling and noxious cooling stimulus, respectively (Leith *et al*., 2010). The neuronal response to water at room temperature (21 °C) was subtracted from the acetone‐evoked and ethyl chloride‐evoked responses to control for concomitant mechanical stimulation during application. Natural stimuli were applied starting with the lowest intensity stimulus with 1 min between stimuli in the following order: electrical, brush, von Frey, cold and heat. Data were captured and analysed by an interface (1401; Cambridge Electronic Design, Cambridge, UK) coupled to a computer with Spike2 software with post‐stimulus time histogram and rate functions.

After three consecutive stable baseline responses to natural stimuli (<10% variation, data were averaged to give control values), 10 nm P‐CTX‐2, purified from Moray eel as previously described (Lewis *et al*., 1991), was injected subcutaneously into the receptive field [30 μL; vehicle: >99% normal saline, 0.05% methanol, 0.1% bovine serum albumin (Sigma)]. A subset of rats was subsequently dosed subcutaneously into the contralateral flank with 30 mg/kg 6‐({2‐[2‐fluoro‐6‐(trifluoromethyl)phenoxy]‐2‐methylpropyl}carbamoyl)pyridine‐3‐carboxylic acid (M8‐An) (synthesized in house; Takeda Cambridge Ltd) [1 mL/kg in 85% normal saline, 10% cremophor (Sigma), 5% dimethylsulphoxide (Sigma)]. For Na_v_1.8 and TRPA1 antagonist studies, 30 μg of A803467 (Tocris, Abingdon, UK) or 30 μg of A967079 (Tocris), respectively, was injected into the receptive field either in isolation [vehicle: 97% normal saline, 2% cremophor (Sigma), 1% dimethylsulphoxide (Sigma)] or co‐injected with 10 nm P‐CTX‐2 (dose volume of 30 μL for all studies). A previous study demonstrated that intraplantar injections of saline do not induce changes in neuronal excitability (Eijkelkamp *et al*., 2013). For all experiments, responses to electrical and natural stimuli were tested at 20 and 40 min post‐injection. One neurone was characterized per rat, and a total of 46 rats were used. At the end of the experiments, rats were killed by isoflurane overdose.

### Statistic

Statistical analyses were performed using SPSS v22 (IBM, Armonk, NY, USA). Heat and mechanical coding of neurones were compared with a two‐way repeated‐measures (RM) anova, followed by a Bonferroni post‐hoc test for paired comparisons. Sphericity was tested using Mauchly's test, and the Greenhouse–Geisser correction was applied if violated. Cold‐evoked, brush‐evoked and electrically‐evoked parameters were compared with a two‐tail paired Student's *t*‐test. All data represent mean ± SEM (*P < 0.05, **P < 0.01, ***P < 0.001).

## Results

### Ciguatoxin‐2 induces cold and mechanical hypersensitivity

**Figure 1:**
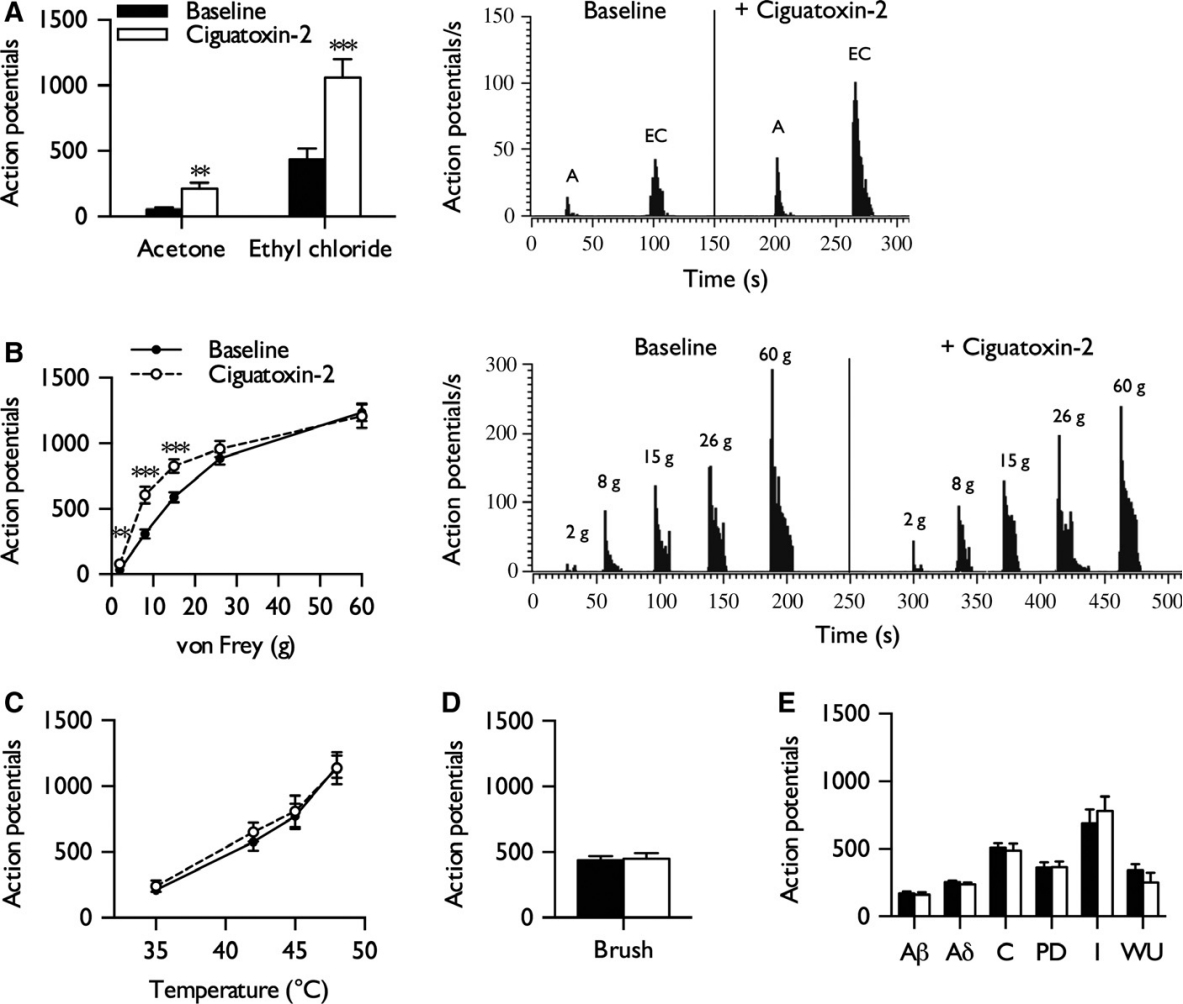
Ciguatoxin‐2 increases neuronal responses to cold and punctate mechanical stimulation. Neuronal responses to cold (A), punctate mechanical (B), heat (C), dynamic brush (D), and electrical (E) stimulation (*n *= 13). Representative traces display single‐unit recordings before and after injection of ciguatoxin. For all natural stimuli, responses were quantified over a 10 s period. After repeated electrical stimulation, action potentials were separated according to latency: Aβ‐fibres, 0–20 ms; Aδ‐fibres, 20–90 ms; C‐fibres, 90–350 ms; post‐discharge (PD), >350 ms. Input (I) and wind‐up (WU) were calculated as described in Materials and methods. Figures display baseline neuronal responses and responses at 20 min post‐injection of 10 nm P‐CTX‐2 into the receptive field; data represent mean ± SEM. Asterisks denote statistically significant difference from baseline: **P < 0.01, ***P < 0.001. A, acetone; EC, ethyl chloride.

The peripheral effects of P‐CTX‐2 were examined in naive rats. Following stable baseline neuronal responses, 10 nm P‐CTX‐2 was injected into the receptive field. P‐CTX‐2 substantially increased neuronal responses to innocuous (acetone) and noxious (ethyl chloride) cold stimulation (paired Student's *t*‐test: innocuous, *P *= 0.0042; noxious, *P *= 0.000025) (Fig. 1A). In addition, P‐CTX‐2 transiently enhanced neuronal responses to punctate mechanical stimuli typically below or around the withdrawal threshold but not to higher noxious forces (two‐way RM anova,* P *= 0.0013, *F*_1,12_ **= 17.468) (Fig. 1B). No effect of the toxin was observed on heat‐evoked (two‐way RM anova,* P *= 0.647, *F*_1,12_ **= 0.222) (Fig. 1C) or brush‐evoked (paired Student's *t*‐test, *P *= 0.86) (Fig. 1D) neuronal responses. After a sequence of supra‐threshold electrical stimuli, the total number of action potentials attributed to Aβ‐fibres, Aδ‐fibres, and C‐fibres did not differ from baseline (paired Student's *t*‐test: Aβ‐fibres, *P *= 0.384; Aδ‐fibres, *P *= 0.119; C‐fibres, *P *= 0.634).

Measures of neuronal excitability, input, wind‐up, and post‐discharge were also unaffected (paired Student's *t*‐test: input, *P *= 0.414; wind‐up, *P *= 0.332; post‐discharge, *P *= 0.930) (Fig. 1E).

### Ciguatoxin‐induced cold hypersensitivity is not reversed by the TRPM8 antagonist M8‐An

**Figure 2:**
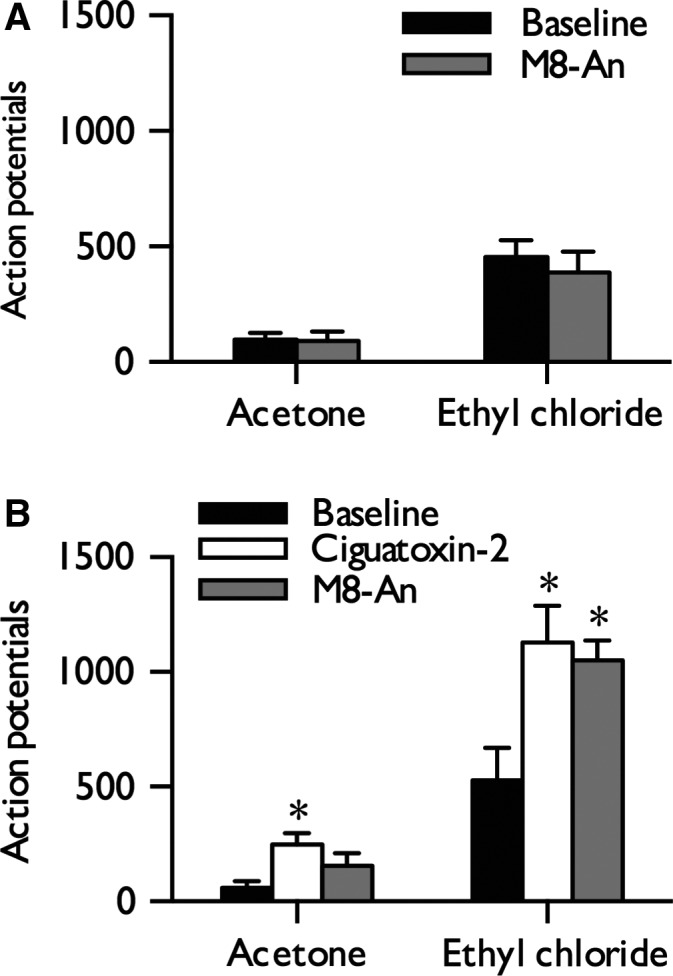
Cold hypersensitivity is not reversed by inhibition of TRPM8. (A) Effect of M8‐An (30 mg/kg) on neuronal responses to innocuous and noxious cold stimulation of the receptive field (*n *= 7). (B) Effect of M8‐An (30 mg/kg) on cold‐evoked responses following the establishment of cold hypersensitivity induced by 10 nm P‐CTX‐2 (*n *= 6). Figures display baseline neuronal responses and responses at 40 min post‐injection of both compounds; data represent mean + SEM. Asterisks denote statistically significant difference from baseline: *P < 0.05.

A novel systemically available TRPM8 antagonist M8‐An has been previously demonstrated to inhibit lamina V/VI neuronal responses selectively to innocuous and noxious cold stimulation in spinal nerve‐ligated rats but not in uninjured rats (Patel *et al*., 2014b). Thus, we examined whether inhibiting TRPM8 would reverse cold hypersensitivity induced by P‐CTX‐2. At an effective dose of 30 mg/kg (delivered subcutaneously), M8‐An alone had no effect on innocuous or noxious cold‐evoked neuronal responses (paired Student's *t*‐test: innocuous, *P *= 0.894; noxious, *P *= 0.258) (Fig. 2A). M8‐An dosed after the establishment of cold hypersensitivity subsequently failed to reduce neuronal responses to innocuous and noxious cold stimulation (one‐way RM anova,* P *= 0.046, *F*_2,10_ **= 4.238) (Fig. 2B).

### Ciguatoxin‐induced cold hypersensitivity is partially inhibited by the TRPA1 antagonist A967079

**Figure 3:**
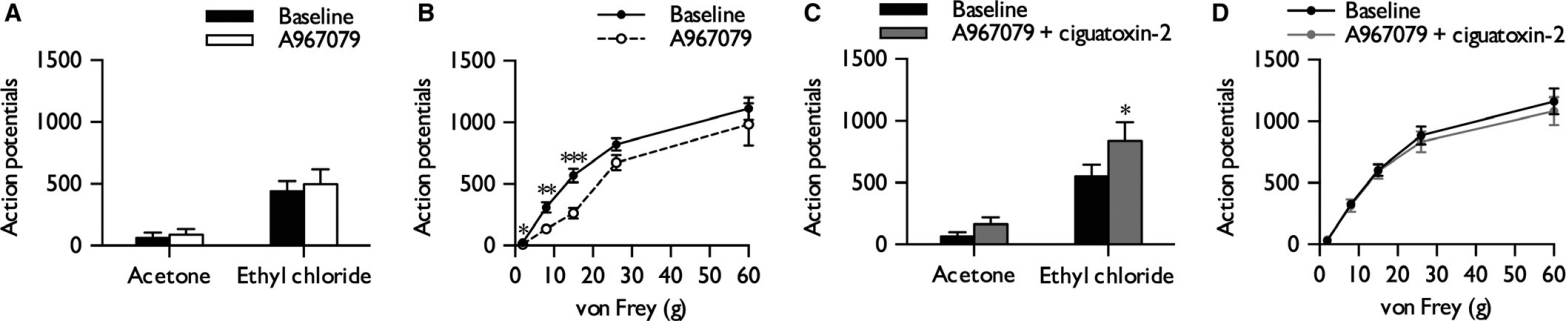
TRPA1 antagonist A967079 partially prevents cold, but not mechanical, hypersensitivity. Effect of A967079 (30 μg) on cold (A) and punctate mechanical (B) stimulation of the receptive field (*n *= 6). Effect of A967079 (30 μg) and 10 nm P‐CTX‐2 on cold (C) and punctate mechanical (D) stimulation of the receptive field (*n *= 8). Figures display baseline neuronal responses and responses at 20 min post‐injection into the receptive field; data represent mean ± SEM. Asterisks denote statistically significant difference from baseline: *P < 0.05, **P < 0.01, ***P < 0.001.

The ability of A967079, a TRPA1 antagonist, to inhibit P‐CTX‐2‐induced hypersensitivity was examined by co‐injecting with 10 nm P‐CTX‐2. An efficacious dose was chosen within the selective range; higher doses were not tested to avoid issues of non‐selectivity (McGaraughty *et al*., 2010). In isolation, A967079 had no effect on cold‐evoked responses (paired Student's *t*‐test: innocuous, *P *= 0.106; noxious, *P *= 0.506) (Fig. 3A), but inhibited neuronal responses to low but not supra‐threshold punctate mechanical stimulation (two‐way RM anova,* P *= 0.0041, *F*_1,5_ **= 25.028) (Fig. 3B). Brush‐evoked and heat‐evoked responses were unaffected (Supporting Information Fig. S1). Co‐injection of A967079 with P‐CTX‐2 only partially blocked the increase in neuronal responses to noxious cooling with ethyl chloride (0.59‐fold increase from baseline compared with 2.46‐fold increase with ciguatoxin alone) (paired Student's *t*‐test, *P *= 0.0155) (Fig. 3C), whereas acetone‐evoked neuronal responses did not statistically differ from baseline (paired Student's *t*‐test, *P *= 0.159) (Fig. 3C). The reduction in mechanically‐evoked responses observed with A967079 alone (Fig. 3B) was no longer apparent when co‐injected with P‐CTX‐2 (two‐way RM anova,* P *= 0.327, *F*_1,7_ **= 1.112) (Fig. 3D).

### Ciguatoxin‐induced cold and mechanical hypersensitivity are inhibited by Na_v_1.8 antagonist A803467

**Figure 4:**
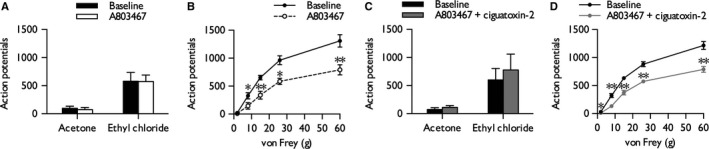
Na_v_1.8 antagonist A803467 prevents ciguatoxin‐induced cold and mechanical hypersensitivity. Effect of A803467 (30 μg) on cold (A) and punctate mechanical (B) stimulation of the receptive field (*n *= 6). Effect of A803467 (30 μg) and 10 nm P‐CTX‐2 on cold (C) and punctate mechanical (D) stimulation of the receptive field (*n *= 6). Figures display baseline neuronal responses and responses at 20 min post‐injection into the receptive field; data represent mean ± SEM. Asterisks denote statistically significant difference from baseline: *P < 0.05, **P < 0.01.

The ability of A803467, an Na_v_1.8 antagonist [>100‐fold selective over related sodium channels (Jarvis *et al*., 2007)], to prevent ciguatoxin‐induced hypersensitivity was examined by co‐injecting with 10 nm P‐CTX‐2. A803467 alone had no effect on cold‐evoked responses (paired Student's *t*‐test: innocuous, *P *= 0.582; noxious, *P *= 0.915) (Fig. 4A), but reduced neuronal responses to low‐intensity and high‐intensity punctate mechanical stimulation (two‐way RM anova,* P *= 0.0045, *F*_1,5_ **= 24.025) (Fig. 4B). Brush‐evoked responses were unaffected although heat‐evoked responses were decreased as previously observed at similar doses (Rahman & Dickenson, 2015) (Supporting Information Fig. S1). Co‐injection of A803467 with P‐CTX‐2 prevented the increase in neuronal response to cold (paired Student's *t*‐test: innocuous, *P *= 0.442; noxious, *P *= 0.363) (Fig. 4C) and mechanical (two‐way RM anova,* P *= 0.0017, *F*_1,5_ **= 56.019) (Fig. 4D) stimulation observed with P‐CTX‐2 alone (Fig. 1A and B).

## Discussion

Here we describe the ciguatoxin‐induced hypersensitivity of spinal neurones. This study demonstrates, for the first time, that ciguatoxins can induce mechanical hypersensitivity in addition to cold hypersensitivity and that the peripheral site of action of the toxin alters the coding properties of spinal sensory neurones. Both cold and mechanical hypersensitivity were prevented by inhibition of Na_v_1.8, whereas TRPA1 inhibition only prevented innocuous cold hypersensitivity and partly prevented noxious cold hypersensitivity at the dose tested; inhibiting TRPM8 had no effect on cold hypersensitivity. Furthermore, we demonstrate, for the first time, that in naive rats inhibiting peripheral TRPA1 and Na_v_1.8 channels does not affect deep dorsal horn neuronal coding to the cooling stimuli applied.

We have recorded from wide dynamic range lamina V/VI neurones whose firing patterns closely correlate with the intensity and temporal aspects of pain in animals and human subjects under normal conditions (Coghill *et al*., 1993; Sikandar *et al*., 2013). In addition, in two parallel animal and human surrogate models of ultraviolet B‐induced peripheral and central sensitization, spinal hyperexcitability in rats correlated with the enhanced perceptual responses of human subjects (O'Neill *et al*., 2015). Hence, these neurones are aptly placed in the sensory pathway to examine neuronal correlates of hyperalgesia. Behavioural cold assays are often hampered by ambiguity, high variability and a lack of overt responses (Yin *et al*., 2015); these neuronal recordings allow consistent responses to multiple modalities of applied stimuli and, furthermore, allow the objective quantification of responses to dynamic brushing and noxious stimuli not amenable to the behavioural testing performed in previous studies (Vetter *et al*., 2012; Zimmermann *et al*., 2013). Numerous lines of evidence have implicated TRPM8 in shaping multiple facets of cold sensitivity in rodents, including a role in hyperalgesia and analgesia (Bautista *et al*., 2007; Dhaka *et al*., 2007; Knowlton *et al*., 2013; Patel *et al*., 2014a,b). However, blocking TRPM8 with M8‐An in naive rats did not appear to reduce spinal neuronal firing to cold stimulation consistent with our previous findings (Patel *et al*., 2014b). M8‐An failed to reverse ciguatoxin‐induced cold hypersensitivity despite being efficacious at this dose in spinal nerve‐ligated rats, indicating distinct differences in the mechanisms of cold hypersensitivity between this peripheral toxin model and that following nerve trauma (Patel *et al*., 2014b). This is supported by previous observations that ciguatoxin‐induced cold hypersensitivity is not diminished in TRPM8 knockout mice (Vetter *et al*., 2012). The evidence from knockout studies for TRPA1 being a transducer of noxious cold temperatures is less than compelling (Bautista *et al*., 2006; Kwan *et al*., 2006; Knowlton *et al*., 2010). Several groups have confirmed the sensitivity of TRPA1 to temperatures below 17 °C (Story *et al*., 2003; Karashima *et al*., 2009; Kremeyer *et al*., 2010), but these findings have been equally disputed with others being unable to reproduce the data (Jordt *et al*., 2004; Nagata *et al*., 2005; Bautista *et al*., 2006). Our data do not support TRPA1 being required for noxious cold sensitivity in naive rats. The tetrodotoxin‐resistant voltage‐gated sodium channel Na_v_1.8 is largely expressed in nociceptors and is localized to nerve endings (Djouhri *et al*., 2003). Na_v_1.8 knockout mice are almost completely unresponsive to noxious cold temperatures. Unlike other sodium channels, the inactivation kinetics of Na_v_1.8 are resistant to cold, identifying Na_v_1.8‐positive neurones as critical for the detection of noxious cold temperatures (Zimmermann *et al*., 2007). Interestingly, the Na_v_1.8 antagonist A803467 does not inhibit noxious cold‐evoked activity in the deep dorsal horn. The ‘channelome’ of cold‐sensitive afferents has been the subject of much debate, but is yet to be clearly defined. Na_v_1.9 frequently co‐localizes with Na_v_1.8, but not TRPM8 or TRPA1, and has recently been identified as a subthreshold amplifier of cold transducer currents. Na_v_1.9 knockout mice exhibit normal temperature discrimination but impaired responses to noxious cooling ramps (Lolignier *et al*., 2015). Sensitivity to low‐threshold cold stimuli, but not noxious cold, is lost in Na_v_1.7‐positive afferent‐ablated mice, whereas the converse is true for Na_v_1.8‐positive afferent‐ablated mice (Minett *et al*., 2014). TREK1/2, TRAAK, TASK‐3, K_v_1 and K_v_7 potassium channels are expressed in cold‐sensitive afferents and are hypothesized to influence activation thresholds to cooling (Madrid *et al*., 2009; Noël *et al*., 2009; Vetter *et al*., 2013; Morenilla‐Palao *et al*., 2014; Pereira *et al*., 2014). The inactivation of background potassium currents results in a net depolarization upon cooling and has been proposed as an alternative mechanism of transduction (Reid & Flonta, 2001). The data presented here support an undefined component of cold sensitivity in rats that may include these mechanisms. In addition, the role of the putative cold transducer TRPC5, gated by cooling between 37 and 25 °C, is not fully understood, although ciguatoxin induces cold hypersensitivity independently of TRPC5 (Zimmermann *et al*., 2011; Vetter *et al*., 2012).

P‐CTX‐2 enhanced neuronal responses to innocuous and noxious cold stimulation in the absence of any obvious changes in central neuronal excitability or coding of other modalities (heat and brush), consistent with a peripheral interaction with cold transduction. *In vitro*, ciguatoxins activate subsets of dorsal root ganglia neurones, including most calcitonin gene‐related peptide‐positive and TRPA1‐positive neurones, and confer cold sensitivity to previously cold‐insensitive TRPA1‐expressing neurones (Vetter *et al*., 2012). Cold hypersensitivity induced by ciguatoxin is dependent on a subset of Na_v_1.8/TRPA1‐positive afferents, which comprise a negligible component of normal cold sensitivity in rats. TRPA1 itself is not directly gated by ciguatoxins (Vetter *et al*., 2012); however, ciguatoxins, in addition to altering the activation/inactivation kinetics of sodium channels, further augment neuronal excitability by inhibition of transient and delayed rectifier potassium currents, the latter of which is sensitive to block by 4‐aminopyridine and influences thresholds for cold sensitivity (Viana *et al*., 2002; Birinyi‐Strachan *et al*., 2005). Membrane depolarization, as a consequence of changes in these ionic conductances, has implications for the voltage dependence of activation for TRPA1 and TRPM8 (Brauchi *et al*., 2004). Despite a substantial co‐expression of Na_v_1.8 in TRPM8‐positive dorsal root ganglia neurones (Shields *et al*., 2012), cold hypersensitivity is dependent on TRPA1 and not TRPM8. This possibly relates to the differential expression of potassium currents hypothesized to determine activation thresholds in low‐threshold and high‐threshold cold sensory neurones (Viana *et al*., 2002). The attenuation of ciguatoxin‐induced cold hypersensitivity by flupirtine, a K_v_7 agonist, does not directly demonstrate that ciguatoxins exert their effects through these channels but would be consistent with this notion (Zimmermann *et al*., 2013). Thus, a cumulative effect on sodium and potassium currents would result in activation of TRPA1 upon cooling but not TRPM8. Rather than being directly gated by cold temperatures, TRPA1 has been proposed to be activated by intracellular calcium during cooling (Zurborg *et al*., 2007), the levels of which are also elevated as a consequence of altered neuronal excitability (Molgo *et al*., 1993).

Although not directly related to this study, perceptually the A‐fibre nerve block, thermal grill and menthol‐induced hyperalgesia model all evoke sensations of paradoxical burning during cooling (Wahren *et al*., 1989; Craig & Bushnell, 1994; Binder *et al*., 2011), and functional magnetic resonance imaging analysis also reveals various degrees of activation within the lateral and medial thalamic nuclei in these models (Binder *et al*., 2007; Lindstedt *et al*., 2011). Similarly, in post‐stroke patients, damage to a cool‐signalling lateral thalamic pathway that causes a disinhibition of a medial thalamic nociceptive channel has been proposed to underlie observed burning cold, ongoing pain and cold allodynia in these patients (Greenspan *et al*., 2004). It is possible that the peripheral and spinal effects induced by ciguatoxins impact on these thalamo‐cortical pathways originating through the spinal changes that we report.

Increased mechanical sensitivity has not been reported clinically; however, P‐CTX‐2 selectively induced a sensitization to low‐threshold punctate mechanical stimuli, which might be dependent on Na_v_1.8‐positive Aδ‐fibre low‐threshold mechanoreceptors and/or C‐fibre low‐threshold mechanoreceptors (Djouhri *et al*., 2003). This was a transient effect, whereas cold hypersensitivity persisted for several hours. This short‐lived effect would explain the disparity between this study and previous observations that ciguatoxins do not induce mechanical hypersensitivity (Vetter *et al*., 2012) as well as the clinical descriptors. Both Na_v_1.8 and TRPA1 have been implicated in mechanosensitivity (Corey *et al*., 2004; Kwan *et al*., 2006; Abrahamsen *et al*., 2008) and the ablation of Na_v_1.8‐positive afferents results in behavioural deficits in mechanical sensitivity (Abrahamsen *et al*., 2008). Here we have shown that the Na_v_1.8 antagonist A803467 reduces mechanically‐evoked neuronal responses to both innocuous and noxious punctate stimuli but not to dynamic brushing. TRPA1 knockout mice display deficits in sensitivity to low‐threshold and threshold levels of punctate mechanical stimulation but not to supra‐threshold stimuli (Kwan *et al*., 2006). This correlates with the neuronal recordings presented here demonstrating that the TRPA1 antagonist A967079 selectively inhibits low‐threshold mechanically‐evoked neuronal responses, whereas noxious‐evoked responses are conserved. Additionally, we observe that A967079 does not affect dynamic brush‐evoked responses, consistent with an absence of TRPA1 expression in Aβ‐fibres (Story *et al*., 2003). A803467 completely blocked any increases in punctate mechanically‐evoked responses when co‐injected with P‐CTX‐2, whereas neuronal responses continued to increase when co‐injected with A967079. Thus, Na_v_1.8 and TRPA1 appear to have distinct modality‐dependent roles in ciguatoxin‐induced hypersensitivity. Activation of TRPA1 may also mediate pruritus (Wilson *et al*., 2013), a prominent symptom associated with ciguatera.

The data presented here corroborate and extend previous *in vitro*, transgenic and functional magnetic resonance imaging approaches in attempting to identify pathways through the pain neuraxis that contribute to the paradoxical sensations experienced upon cooling in ciguatera (Vetter *et al*., 2012; Zimmermann *et al*., 2013). Peripheral neuropathies such as chemotherapy‐induced and diabetic neuropathy‐induced cold allodynia are thought to depend in part on disturbances in ionic conductances in primary afferent fibres (Descoeur *et al*., 2011; Bierhaus *et al*., 2012) and may share overlapping mechanisms with ciguatera but differ from mechanisms of cold hypersensitivity after nerve trauma.

## Disclosures

The authors have no conflicts of interest to declare.

## Supporting information

Fig. S1. Effect of Na_v_1.8 antagonist A803467 (30 μg) on brush (A) and heat (B) evoked neuronal responses (*n*=6). Effect of TRPA1 antagonist A967079 (30 μg) on brush (C) and heat (D) evoked neuronal responses (*n*=6). Figures display baseline neuronal responses and responses 20 minutes post‐injection into the receptive field; data represent mean ± SEM. Asterisks denote statistically significant difference to baseline, *P < 0.05.
