# Supplementary figures and images for: Ionic mechanisms of spinal neuronal cold hypersensitivity in ciguatera
Source: Eur J Neurosci. 2015 Nov 13;42(11):3004–11. doi: 10.1111/ejn.13098 (PMC4744673; doi:10.1111/ejn.13098)

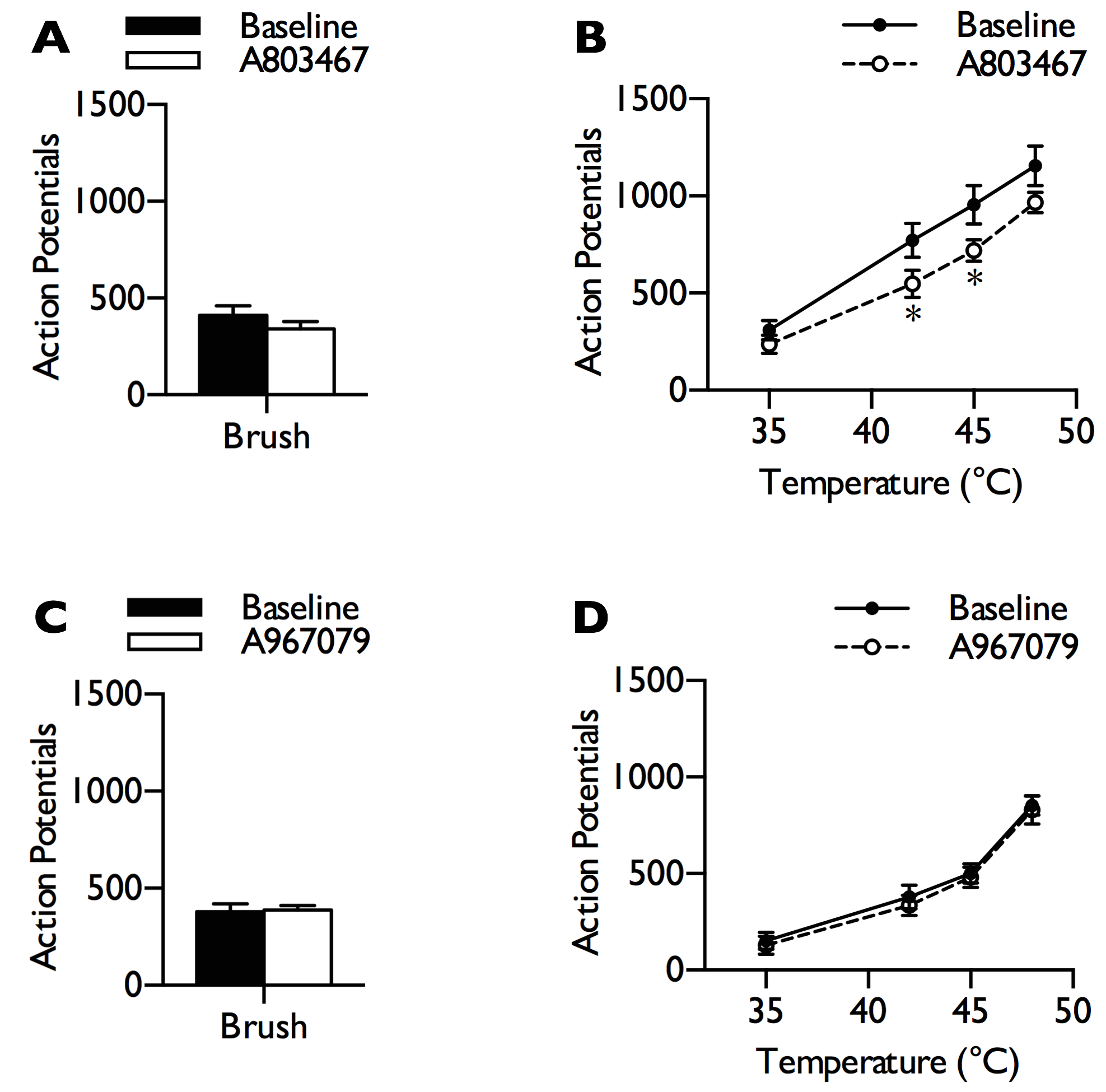

Supplement: Supplementary file 1 — Fig. S1. Effect of Nav1.8 antagonist A803467 (30 μg) on brush (A) and heat (B) evoked neuronal responses (n=6). Effect of TRPA1 antagonist A967079 (30 μg) on brush (C) and heat (D) evoked neuronal responses (n=6). Figures display baseline neuronal responses and responses 20 minutes post‐injection into the receptive field; data represent mean ± SEM. Asterisks denote statistically significant difference to baseline, *P < 0.05. [file EJN-42-3004-s001.tiff]
